# Monitoring Adherence Rate to Growth Hormone Therapy and Growth Outcomes in Taiwanese Children Using Easypod Connect: Observational Study

**DOI:** 10.2196/14774

**Published:** 2021-01-15

**Authors:** Pen-Hua Su, Chen Yang, Mei-Chyn Chao, Chung-Lin Chiang

**Affiliations:** 1 School of Medicine Chung-Shan Medical University Taichung City Taiwan; 2 Department of Pediatrics and Genetics Chung Shan Medical University Hospital Taichung City Taiwan; 3 Division of Genetics, Metabolism and Endocrinology Department of Pediatrics Taipei Medical University Hospital Taipei Taiwan; 4 Department of Pediatric Genetics Changhua Christian Children's Hospital Changhua Taiwan; 5 Department of Pediatric Genetics, Endocrinology and Metabolism Kaohsiung Medical University Hospital Kaohsiung Taiwan; 6 Merck Ltd. Taipei Taiwan

**Keywords:** growth hormone, adherence, easypod, eHealth

## Abstract

**Background:**

Adherence to growth hormone therapy is difficult to detect reliably. Devices such as easypod have been developed for electronic recording of injections. The easypod connect observational study (ECOS) was an open-label, observational, multinational, phase IV study conducted in 24 countries around the world. The final results from ECOS in the Taiwanese cohort are reported in this paper.

**Objective:**

This study aimed to evaluate the adherence and long-term outcomes of growth hormone therapy in pediatric subjects using the easypod electromechanical device.

**Methods:**

Subjects (aged 2-18 years or >18 years without fusion of growth plates) who received Saizen (recombinant human growth hormone, somatropin) via the easypod device were enrolled in this study. The primary objective was to assess the level of adherence in subjects receiving Saizen via easypod.

**Results:**

In Taiwan, a total of 35 and 13 children fulfilled the criteria of full analysis set and complete analysis set, respectively. The mean (SD) age of the complete analysis set was 12.08 (2.72) years. All subjects were growth hormone–naïve, with 38% (5/13) females. The mean adherence rates of 13 subjects were 87.6% at 3 months and 84.3% at 6 months, that of 8 subjects was 81.0% at 9 months, and that of 4 subjects was 91.6% at 1 year. After 1 year of treatment, subjects had a median (Q1:Q3) change in height SD score of 0.30 (0.06:0.48), median height velocity of 6.50 (4.33:8.24) cm/year, and median change in height velocity SD score of 1.81 (–0.04:3.52).

**Conclusions:**

With the easypod device, patients with inadequate adherence and poor response to treatment can be identified. Adherence to growth hormone therapy administered via easypod was generally high in the first year of treatment but the adherence gradually decreased over time. Overall, growth outcomes after 1 year indicated a positive growth response to growth hormone treatment. Future efforts should be focused on personalized management of adherence by using the easypod system.

## Introduction

### Background

Human growth hormone, also known as somatotropin, is synthesized and secreted by the somatotropic cells of the anterior pituitary gland and it plays a critical role in growth and metabolism. Recombinant human growth hormone was first approved for the treatment of childhood growth hormone deficiency in 1985 [1]. Since then, synthetic human growth hormone has been widely administered for the treatment of inadequate secretion of endogenous growth hormones in children and adults. For pediatric patients, growth hormone is indicated for treating growth disorders due to a number of medical causes, including growth hormone deficiency, Turner syndrome, and children born small for gestational age. During the past several decades, growth hormone therapy has demonstrated its effects on improving growth outcomes and helping children achieve catch-up growth [2-5].

### Adherence to Growth Hormone Treatment

As growth hormone therapy for children generally starts at a young age and lasts for several years, both the child and the family are involved in this long-term treatment process. For chronic non–life-threatening conditions such as growth hormone deficiency [6], adherence to treatment is relatively difficult to maintain at a high level, especially when the benefits are not immediately apparent, and regular subcutaneous injections with a frequency of up to once daily causes both physical and psychological burdens. Even though adherence can be monitored through methods such as diary cards or by comparing total expected growth hormone usage to the total amount of growth hormone prescribed, the data could easily be overestimated and become unreliable since the child or the parents may be reluctant to admit missing injections [7].

Studies have shown that growth outcomes of growth hormone therapy could be affected by multiple factors [8-10], among which, poor adherence is still a major problem in treating growth disorders for pediatric patients [11,12]. Although the results vary substantially between studies due to the methods and definitions applied, a prevalence of 5%-82% has been reported [13]. Poor adherence not only results in suboptimal growth but also increases unnecessary medical expenses [14,15].

The frequency of inadequate adherence is usually underestimated when assessed using conventional methods (eg, diary cards, questionnaires, number of returned vials) [13,16], which only give fragmentary pictures of a patient’s dosing history. In addition, the aforementioned methods cannot completely reveal the patterns of nonadherence such as reduced dosage, drug holiday, or delayed initiation [16]. Thus, electronic monitoring of drug dosing histories is currently recognized as a standard for adherence quantification [16]. The electronic monitoring of injections via devices such as easypod provides information on how many doses have been taken as prescribed and about nonadministered doses, thereby reflecting the extent to which the patient is adherent to the therapy. Through adequate monitoring methods, physicians are able to promptly evaluate the adherence following an inadequate response to growth hormone therapy [17,18].

### Objectives

The easypod connect observational study (ECOS) was an open-label, observational, longitudinal study conducted in 24 countries (Argentina, Australia, Austria, Canada, China, Colombia, Czech Republic, Finland, France, Greece, Hungary, Indonesia, Italy, Kingdom of Saudi Arabia, Korea, Mexico, Norway, Singapore, Slovakia, Spain, Sweden, Taiwan, United Arab Emirates, and the United Kingdom) with a total of 1203 subjects included for analyses. It aimed to evaluate the adherence and long-term outcomes of therapy in pediatric subjects using the easypod electromechanical device for growth hormone treatment and to undertake population-based analyses to generate hypotheses relating to drivers of individual adherence [19]. The results of the ECOS have been published by Koledova et al [19]. Among the countries involved, the results of Spain [20], Italy [21], and Mexico [22] have been published. However, there is no related publication in the Asia-Pacific region. The culture and living habits might possibly influence medication adherence. Clinically, some children in Taiwan go to bed late, resulting in late administration of the growth hormone, which may indirectly affect adherence. Therefore, in this study, we present the results of Taiwanese pediatric subjects.

## Methods

### Study Design

ECOS was a multinational, multicenter, observational, longitudinal, open-label, phase IV study conducted between November 2010 and February 2016. The study was conducted in accordance with principles of the Declaration of Helsinki and the protocol, as well as the good clinical practice (ICH-GCP E6) and the applicable national legal and regulatory requirements. The study protocol was approved by the institutional review board at each study site, and written informed consent or assent was obtained from all subjects’ parents or legal guardians before enrolment.

### Patients

Subjects (aged 2-18 years or >18 years without fusion of growth plates) who received Saizen (Merck KGaA) via the easypod electromechanical device were enrolled. Subjects who were receiving growth hormones in whom growth plates had fused (ie, for taking growth hormones for its metabolic effects), subjects with contraindications to Saizen as per locally approved prescribing information, subjects using an investigational drug, or subjects participating in an interventional clinical study were excluded from the study. The duration of follow-up for growth hormone treatment was planned to be at least 6 months and up to 5 years. There was 1 baseline visit followed by 1-4 subsequent visits per year as per routine practice. All assessments were performed during the visits. As an observational study, growth hormone treatment and other aspects of patient management were entirely at the discretion of the physician and his or her patient, following a standard clinical practice.

### Data Collection and Study Endpoints

The primary endpoint was treatment adherence rate (percentage of prescribed injections that were administered) over time. Data on injection time, date, dose, planned frequency were uploaded to a secure web-based database via a specific connection kit and the physician’s computer. For subjects who had consented to participate in the observational study, deidentified data were then uploaded to the web-based registry/observational study. While adherence data from the enrolled subjects were primarily derived from the easypod device, other information such as demographics, relevant medical and treatment history, and auxological data (eg, height, growth velocity, and bone age) were entered by the physician into the electronic case report form. Data were collected at every visit, as available per routine practice.

### Statistical Analysis

Analysis sets included a full analysis set and a complete analysis set. The full analysis set consisted of all the subjects included in the study, whereas the complete analysis set consisted of all the subjects of the full analysis set without missing the treatment start date on the electronic case report form, without gap in the injection information of more than a week after the start of the treatment, and with height measurement closest to the treatment start date not missing using a window of 3 months (91 days). All statistical analyses on adherence rates were performed on the complete analysis set and were performed in a descriptive way for the endpoints, considering this was a single-arm, noninterventional study. Continuous variables were described with the number of subjects, number of subjects with missing data, mean (SD), median, first and third quartiles (Q1, Q3), and minimum and maximum values. For categorical variables, summary statistics were the number and percentage of subjects in each category. To calculate height standard deviation score (SDS) and height velocity (HV) SDS, the reference median growth parameter and the SD of the reference growth parameter were applied. The World Health Organization reference growth table [23] and the Tanner and Whitehouse reference growth table [24] were used for height SDS and HV SDS derivation, respectively.

## Results

### Patient Characteristics

The ECOS was conducted in 3 medical centers in Taiwan. A total of 35 children had sufficient data and were included in the full analysis set, of which 13 subjects fulfilled the criteria of the complete analysis set. Among the 35 subjects of the full analysis set, 32 had growth hormone deficiency, 2 were born small for gestational age, and 1 had Turner syndrome. The average age was 12.26 years. More than half of the subjects were male (19/35, 54%). At baseline, all subjects were growth hormone–naïve, with a mean height of 137.06 cm and a mean growth velocity of 4.14 cm/year. The baseline demographic characteristics and auxological data are shown in Table 1.

**Table 1:** Demographic and auxological data of the subjects at baseline.

Demographic and auxological data		Full analysis set (N=35)	Complete analysis set (n=13)
Age (years) (min, max)		12.26 (7.0, 16.0)	12.08 (6.0, 16.0)
**Sex, n (%)**			
	Female	16 (46)	5 (39)
	Male	19 (54)	8 (61)
Asian ethnicity, n (%)		35 (100)	13 (100)
**Pubertal stage, n (missing)**		21 (14)	5 (8)
	Tanner 1, n (%)	4 (19)	0
	Tanner >1, n (%)	17 (81)	5 (100)
**IGF-1 status, n (missing)**		10 (25)	2 (11)
	Abnormally low, n (%)	0	0
	Normal, n (%)	8 (80)	2 (100)
	Abnormally high, n (%)	2 (20)	0
Bone age, n (missing)		15 (20)	9 (4)
Greulich and Pyle assessment (years), (min, max)		11.84 (3.0, 15.7)	10.72 (3.0, 15.0)
Growth velocity (cm/year), (min, max)		4.14 (0.0, 9.4)	3.05 (0.0, 4.9)
Height (cm), (min, max)		137.06 (103.0, 161.0)	139.26 (103.0, 161.0)
**Indication for growth hormone treatment, n (%)**			
	Growth hormone deficiency	32 (91)	11 (84)
	Small for gestational age	2 (6)	1 (8)
	Turner syndrome	1 (3)	1 (8)
	Other	0	0
Adjusted mid-parent’s height (cm), (min, max)		162.79 (151.0, 179.0)	164.55 (153.0, 179.0)

### Adherence Rates of the Subjects

**Figure 1:**
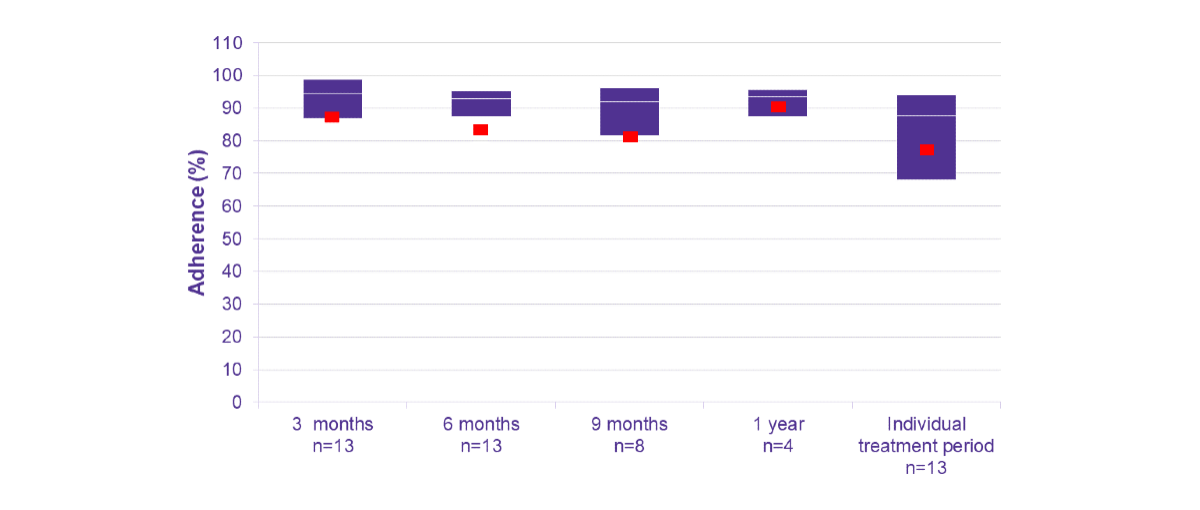
Treatment adherence rates over time (complete analysis set). Boxes show Q1 and Q3, with median as white line and mean as red squares.

**Figure 2:**
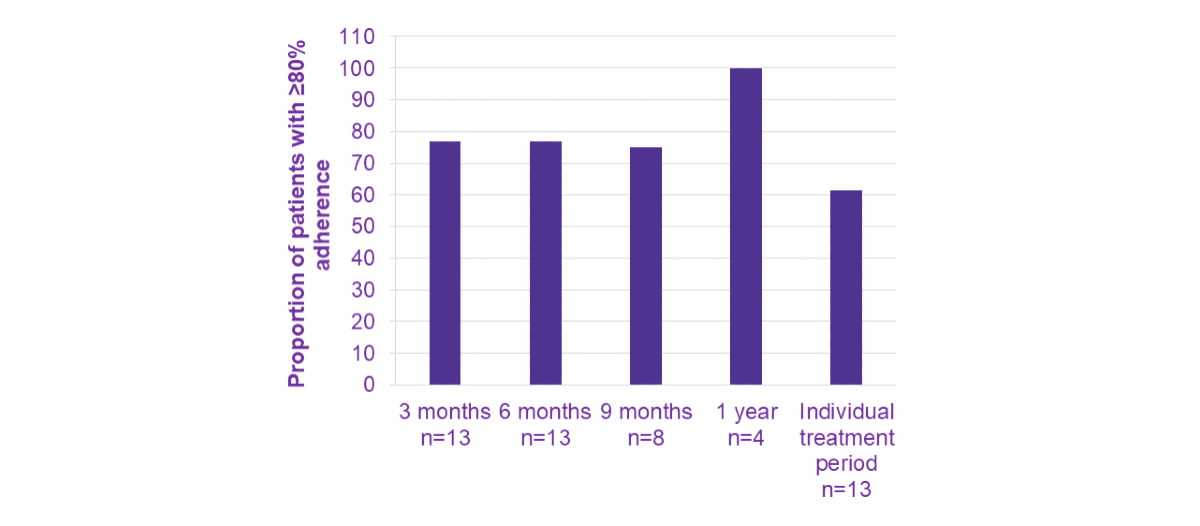
The proportion of patients treated with growth hormone using easypod with adherence rates of at least 80% over time and for all patients at any time within the study period.

**Figure 3:**
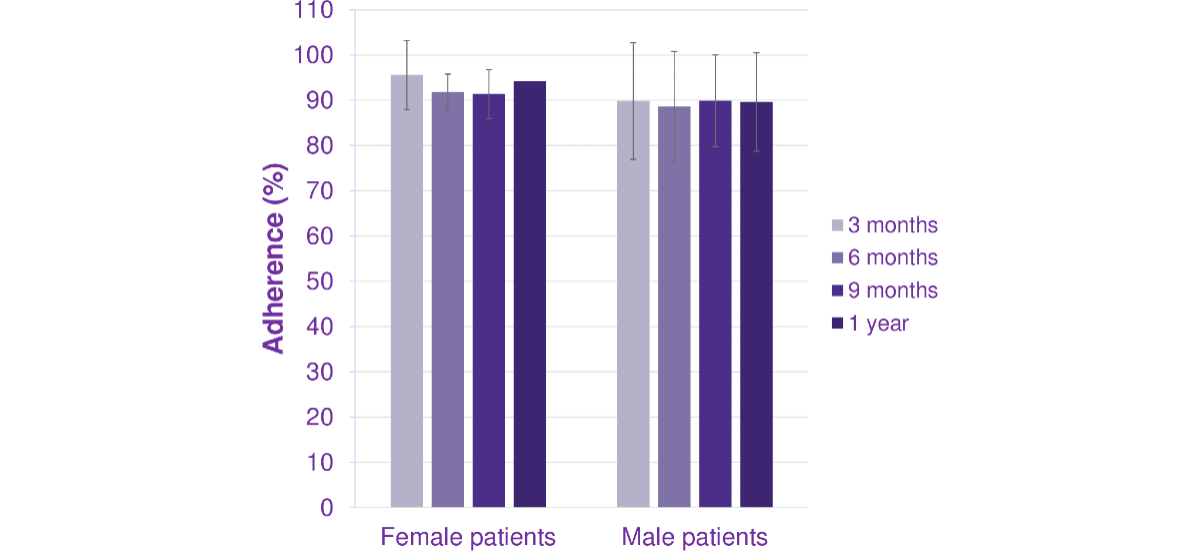
Treatment adherence rates over time by gender (complete analysis set).

The primary endpoint was the adherence rate of subjects receiving Saizen via easypod over a period of time. Among the 13 subjects in the complete analysis set, the longest follow-up period was approximately 1.5 years, with a mean (SD) treatment duration of 332 (113.1) days, and the proportions of subjects with adherence data available for 3, 6, 9 months, and 1 year were 100% (13/13), 100% (13/13), 62% (8/13), and 31% (4/13), respectively. The median (IQR) of adherence rates over increasing periods of follow-up are presented in Figure 1. The mean adherence rate was 87.6% at 3 months, 84.3% at 6 months, and 81.0% at 9 months, indicating a slight decrease in adherence rate over time. The mean adherence rate was calculated by averaging all patients’ adherence rates during a period of time. The majority of the complete analysis set subjects maintained an adherence rate of ≥80%, and these percentages remained steady at 3, 6, and 9 months (Figure 2). Subgroup analysis by sex revealed that the median adherence rates were similar between the female and male subjects (Figure 3).

### Growth Outcomes of the Subjects

After 1 year of treatment, subjects had a mean (SD) change in height of 6.25 (3.07) cm, height SDS of +0.27 (0.30), mean (SD) HV of 6.49 (2.95) cm/year, and mean (SD) change in HV SDS of 1.51 (2.22). The growth outcomes and changes from baseline after 1 year of growth hormone treatment are summarized in Table 2. Spearman product-moment correlations between these outcomes and adherence rates were assessed to further investigate the impact of adherence on growth outcomes. Nevertheless, limited by the number of subjects with available data (n=4), no significant and consistent correlation was identified in the complete analysis set (data not shown). Overall, growth outcomes after 1 year indicated a positive growth response to growth hormone treatment.

**Table 2:** Growth outcomes and changes from baseline of the subjects after 1 year of growth hormone treatment using easypod (complete analysis set).

Growth outcome	Subjects with growth hormone deficiency (n=11)	Subject who was small for gestational age (n=1)	Subject with Turner syndrome (n=1)	Overall (n=13)
Baseline height (cm), mean (SD)	141.95 (14.32)	103.00	146.00	139.26 (17.05)
Change in height (cm), mean (SD)	6.20 (3.24)	8.50	4.50	6.25 (3.07)
Baseline height, SDS^a^ (SD)	–1.74 (0.94)	–2.68	–2.38	–1.86 (0.91)
Change in height SDS at 1 year, mean (SD)	0.22 (0.29)	0.59	0.56	0.27 (0.30)
Baseline height velocity (cm/year), mean (SD)	3.30 (1.17)	3.92	0	3.05 (1.47)
1-year height velocity (cm/year), mean (SD)	6.56 (3.12)	7.92	4.33	6.49 (2.95)
1-year height velocity SDS, mean (SD)	1.45 (2.31)	2.23	—^b^	1.51 (2.22)

^a^SDS: standard deviation score.

^b^Not available because of missing data.

## Discussion

The ECOS assessed the adherence to recombinant human growth hormone treatment as well as growth outcomes in pediatric patients with growth disorders. The results of the European and American countries involved in this open-label, observational, longitudinal study have been published [20-22]. To our knowledge, there is no literature exploring the adherence to growth hormone treatment in Taiwanese or Chinese pediatric patients in a real-life setting, especially by using an electronic monitoring method. In Taiwan, physicians’ clinical experience has shown that some children have relatively low adherence owing to late administration of growth hormones with late bedtime. The culture and living habits of Asians such as children’s daily routines, the time of going to and coming from school, and the activities after school are quite different from others in the world. The medication adherence might be possibly influenced by these differences. In this study, the Taiwanese cohort of the ECOS is reported. The mean adherence rate was generally high in the first year of the treatment, with the majority of the complete analysis set subjects maintaining an adherence rate of greater than 80%. Although the adherence gradually decreased with a longer duration of follow-up, it is in line with the global ECOS results [19] as well as with that of previous studies showing that the adherence rate diminished over time [15,25].

Reduced adherence to growth hormone therapy is detrimental to therapeutic outcomes [11,14,26] and is considered one of the major causes of suboptimal growth [27]. To maximize the effect of growth hormone therapy, it is necessary to maintain good adherence throughout the entire treatment course. Nonadherence not only represents an obstacle to effective treatment, but it also leads to an increase in the medication costs from direct and indirect aspects [13]. Previous literature has shown that adherence could be negatively or positively associated with a variety of factors such as reduced HV [13], comprehensive medical education/training [28], duration of treatment [10,26,29], and choice of injection device [29]. Through real-time monitoring, timely interventions can be prompted in response to nonadherence, rather than signaled by suboptimal growth at a later stage. Moreover, with reliable information regarding adherence at hand, physicians are able to tell whether suboptimal growth arises from nonadherence or other possible causes.

The prevalence and the level of adherence rate vary considerably among studies, which is partly attributable to the methods applied as well as inconsistent definitions used across the studies [13]. While it has been reported that 39%-66% of the patients missed more than 1 injection per week [10,14], another study showed that the median adherence rate might be up to 95% [30]. Most previous studies investigating adherence were cross-sectional, and the adherence was assessed with a questionnaire-based survey [28,29,31]. As an electronic monitoring device is currently recognized as a standard for quantifying adherence [16], it provides information of the precise time and doses of injections, which allows further analyses for nonadherence patterns [16]. A concordance of 84.3% between adherence reported by patients and recorded using easypod has been demonstrated in a study, and the authors found that there was a trend toward self-reported adherence being higher than the recorded adherence [7]. Nevertheless, it is not known whether the difference resulted from forgetfulness, fear of disappointing practitioners, or a combination of factors [32], and no data are currently available to assess this supposition.

In this study, one of the study objectives was to describe the impact of adherence on clinical outcomes for subjects receiving Saizen via easypod. In fact, with 1190 evaluable subjects, the ECOS global results revealed that statistically significant correlations of 0.13 and 0.08 were observed between adherence rate and change in height SDS and between adherence rate and HV SDS, respectively, indicating a positive correlation between adherence rate and growth outcomes. Unfortunately, the number of subjects was not sufficient to support such analyses for the subgroup of Taiwanese patients, since only 4 patients were administered Saizen for more than 1 year. To consolidate the correlation between adherence and growth outcomes, larger sample sizes are required for future studies.

Suboptimal adherence is a common problem in growth hormone treatment. Since adherence to growth hormone therapy is critical for the optimization of treatment outcomes, it has to be taken into account while evaluating the therapeutic effects for treatment modulation in routine clinical practice. Detection of nonadherence can be difficult using pre-electronic monitoring methods because the patient may be reluctant to admit such behavior [7]. The electronic monitoring of injections via devices such as easypod provides reliable and objective information on how many doses have been taken as prescribed and about the nonadministered doses, which reflect the extent to which the patient is adherent to the therapy. The electronic monitoring device, as distinct from conventional monitoring methods, is less labor-intensive and enables physicians to review the timing, date, and dosage of recombinant human growth hormone delivered in a real-time manner. It may help promote adherence and prompt disease management for routine practice.

This study was restricted by its observational nature as there was a considerable level of missing data and intersubject variability. In addition, as mentioned above, the number of subjects included in the complete analysis set was also limited, and most patients had a treatment duration of less than 1 year. Nevertheless, this paper shows the adherence patterns of pediatric patients using an electronic monitoring device, which have not been previously reported in a Taiwanese patient population. To the best of our knowledge, this is the first study providing insight into the adherence rate and characteristics of Taiwanese pediatric patients who require growth hormone treatment. Of note, future studies are warranted to confirm the results and to further explore the effects of individual variables such as bone age at baseline, socioeconomic statuses, and parental, marital, or employment status.

This was a phase IV, open study, and its conditions were different from the phase II or phase III randomized controlled trial. As a phase IV postmarketing study, it generally aims to explore treatment effectiveness and long-term safety. Compared with a randomized controlled trial, observational trials usually reflect the actual clinical treatment effectiveness because the trial design does not have as many limitations in the inclusion/exclusion conditions as a randomized controlled trial. This study did not specify the length of time of patients receiving easypod treatment. Many patients were treated for less than 1 year. In addition to being limited by the number of results, the length of the treatment period is also one of the possible reasons.

Collectively, this study unveils the adherence over time among Taiwanese pediatric patients receiving growth hormone treatment via the easypod electronic monitoring device. The growth outcomes and changes after 1 year of treatment are also presented, although the associations between adherence rate and growth outcome as well as factors affecting adherence to growth hormone therapy in Taiwanese patients were limited by the sample size. The electronic monitoring/injection device serves as a useful tool for both patients and physicians to help disease management and provide direct information regarding adherence to growth hormone therapy.

## Abbreviations

ECOS: easypod connect observational study
HV: height velocity
SDS: standard deviation score
